# Progress of Surgery

**Published:** 1901-07-13

**Authors:** 


### Progress of Surgery.

(*Continued from page 232.*)

**Movable Kidney.**—Robert T. Morris,[3] of New York, has described a method of fixation for movable kidney, and discussed the symptomatology of the affection. Guyon first suggested removal of part of the fibrous capsule of the kidney, believing that the lymph-exudate from bare parenchyma would make a firm anchorage for the sutured kidney. This method and its modifications have given excellent results at the hands of some operators, but not wholly satisfactory results with others. Some operators have removed the fatty capsule almost completely, and very little of the fibrous capsule; others have removed almost all the fibrous capsule and little of the fatty; some have sutured the kidney in its normal position, others well up into the muscle incision, while others have sutured it in a lower position than normal, believing correctly that it is not the position of the kidney but the influence of its excursions that causes trouble. Senn introduced the method of scarifying the fibrous capsule, and then packing iodoform gauze about the kidney until granulation tissue was well formed. Morris has adopted this plan successfully in two cases, but the time required for repair he found was too long, and the scar too evident. Morris has observed both at subsequent operations and at post-mortem examinations made years later, that replacement of a considerable part of the parenchyma by fibrous tissue had followed in some cases when sutures had been passed through the renal parenchyma. The method the author describes is not original. He found it mentioned in a foreign journal, but has been unable to recall the name of the surgeon who suggested it. The operation consists in turning up a flap of the capsule of the kidney, including the larger part of the mesial surface, raising it towards the convex border of the organ, drawing it through a slit in the psoas or quadratus muscle and suturing it there. Bare parenchyma comes into contact with the psoas or quadratus fascia. He has had very good results from this operation. Clinically, the author groups the cases into (1) those in which there are no troublesome symptoms; (2) those in which there are distressing symptoms; and (3) cases occurring as a part of a general enteroptosis. The cases of the second class suffer from three kinds of symptoms; those of pressure on the bile duct; those of pressure on the duodenum; and those of pressure on the superior mesenteric vein. In the first class there may be obstructive jaundice and the symptoms resemble those of gall-stones. In the second class the symptoms are due to gastric disturbances of various degrees. In one case Morris performed gastroraphy after fixing the kidney, as the stomach was greatly dilated. In the third group the pressure in the superior mesenteric vein causes venous stasis and congestion in the region of the appendix and cæcum. These latter cases had usually been sent to the author for operation upon the appendix. But movable kidney more commonly produces reflex disturbances than direct pressure complications. Morris has seen one case where the effect was a spasm of the œsophagus, for which surgeons had proposed various operations. The symptoms in this class may be referable to the heart, or stomach, or there may be functional neurosis of the intestine. Morris has met with several cases of membranous colitis due in this way to movable kidney, and he quotes one instructive example. The cases of movable kidney associated with general enteroptosis are in many cases treated satisfactorily without operation. C. Knox Shaw, of London,[4] has recorded an interesting case of nephropexy. The patient, a woman of 35 years of age, had for 10 years been liable to attacks of pain in the left side of the abdomen. Both kidneys were prolapsed. On repeated examination it was found that sometimes a large mobile elastic swelling was felt in the left side of the abdomen, but at other times this was absent. The swelling was taken to be the swollen left kidney. The left kidney was therefore fixed by operation. Four days before leaving hospital she had one of her old attacks, and the swelling reappeared. Some months later, as the attacks continued, she was anæsthetised for an exploratory operation. When examined, however, the swelling had disappeared. Suddenly during the examination the right kidney descended and passed to the left side. Thus the tumour was proved to be the right kidney, and that organ was accordingly fixed. The method of operation preferred by Shaw is one based on that of Senn as modified by Deaver. The fatty capsule of the kidney is only removed posteriorly. A pair of forceps is passed through the anterior part of the fatty capsule on each side of the hilum and two long strips of sterilised gauze drawn through. The surface of the kidney is scarified, and pieces of gauze are packed in the wound inside the two long strips. The latter are then tied over the packing and thus removed all chance of the kidney slipping.

**Physical Diagnosis in Kidney Disease.**—David Newman, of Glasgow,[5] has written on this subject. He points out that, viewed from the front, the kidneys, in a normal condition, occupy a position within a rectangular figure bounded below by a horizontal line drawn through the umbilicus, above by a similar line at the level of the ensiform cartilage, and laterally by perpendicular lines 4 inches to the right and left respectively of the middle line. Posteriorly the boundaries may be fixed by drawing the upper horizonal line at the level of the uppermost point of the spinous process of the eleventh dorsal vertebra, the lower at the lower edge of the spinous process of the third lumbar vertebra, and the lateral lines vertically at a distance of 3¾ inches to the right and left of the middle line. Referring to the method of examining the kidneys by *ballottement* he states that by it the observer, with a little practice, may be enabled to appreciate an increase in size of the kidney, and its form and consistence, at a time when ordinary palpation may not show much change in the bulk or form of the gland. Ballottement is a refined method of palpation to which M. Gayon called attention in 1886. It is carried out with the patient lying on the back and breathing freely with the abdominal muscles relaxed. The surgeon uses his two hands. The fingers of the hand in the lumbar region are pressed gently forwards and the patient asked to rest upon them without straining. With the other hand the anterior abdominal wall is slightly pressed backwards—enough to diminish the space separating it from the kidney, but not sufficiently to allow the two to come in contact. The surgeon then gives a short sudden jerk from behind, and as he does so the kidney, if increased in size or unduly mobile, is projected forwards and lightly touches the anterior abdominal wall. The jerks given from behind must be abrupt, so that the muscles may be taken by surprise, and the pressure must be sustained until the kidney touches the anterior abdominal wall. The movement is not to be taken as evidence that the kidney is unduly mobile, for it can often be obtained when there is enlargement only.

**Kidney Insufficiency and Operative Treatment.**—Kümmell[6] has given an account of some experiments and clinical observations which should be of use to operating surgeons in estimating the functional efficiency of the kidneys. The present methods of obtaining this knowledge consist in (1) estimating the amount of urea excreted, (2) finding the freezing point of the blood, of the urine in the bladder, and the urine collected from the ureters. Insufficiency may be diagnosed if the daily output of urea is less than 16 grains. The freezing point of blood is from 0·55° to 0·58° C. A rabbit in which the kidneys were extirpated had a blood freezing point of 0·56° C. before operation, and twenty-four hours after of 0·64° C. A patient who died from anuria after removal of an ovarian carcimona had the blood freezing point of 0·65° C. An animal, some time after removal of one kidney had a normal blood freezing point, showing perfect compensation had been provided by the other kidney. The freezing point of the bladder urine varies within the large limits of 0·9° C. and 2° C. If this freezing point becomes lower than 0·9° C. then removal of any kidney substance is contraindicated. The freezing points of the blood and of the urine, taken in conjunction, will yield valuable evidence as to the degree of renal sufficiency. The term "cryoscopy" is applied to these procedures. In this connection the phloridzin test (Castron) of renal sufficiency may be alluded to. When phloridzin is given each kidney converts it into glucose, and eliminates the glucose in a quantity proportionate to the working integrity of the organ; in other words the affected organ will emit less sugar. It will be noticed that ureteral catheterisation is required in applying both the phloridzin test and that of cryoscopy when the functional efficiency of each kidney has to be ascertained.

**Post-operative Anuria.**—F. Tilden Brown, of New York,[7] has recorded a case of this kind, and commented upon it in an instructive manner. The case was that of a youth of nineteen, upon whom the operation of nephrotomy for pyorrphrosis had been performed nine months previously, and a persistent sinus had remained. At times the sinus almost closed, and painful overdestension of the kidney resulted. It was decided to remove the affected left kidney. This decision was made after the ureters had been catheterised and the right kidney had in that way been proved to secrete normal urine. The kidney was found after removal to be typically pyonephrotic, and to present no tuberculous or calculous formations. After the operation the patient was given alternately nitroglycerine and strychnine for thirty-six hours. During the first six hours 4 ozs. of urine were passed, but during the remaining forty-eight hours he lived no urine was passed. Vomiting became frequent and the heart gradually failed. Saline intra-venous injections were given during the last twelve hours and the nitroglycerine stopped. At the autopsy fatty degeneration of the heart was proved to exist. The remaining kidney showed congestion but no nephritis. Failure of a fatty heart was the cause of death. What made it fail? Was the cause general systemic shock or was it inhibition due to auto-infection in consequence of renal failure? The following causes may have led to the congestion of the kidney:—(1) Chloroform anæsthesia; (2) Compression of the kidney against the spine when sand bags were placed under the flank; (3) Reflex vasomotor effects, the result of ganglionic pressure or of removal of the other kidney; and (4) A congestion incidental to an increased physiological demand. Brown takes the view that the symptoms shown by the patient were those of the erethistic type of shock, and that this so lowered the renal pressure that passive congestion of the kidney and anuria resulted. In choosing chloroform rather than ether as the anæsthetic, Brown was influenced by Kamp's. deductions that ether lowers renal pressure though it raises the general blood pressure, and that chloroform has little effect upon renal pressure. Other workers do not agree with these views; Gallcazi and Grillo, for instance, maintain that chloroform diminishes renal permeability. Brown thinks the chloroform added to the pre-existing heart weakness, and that it would have been much better to have used gas and ether. The author suggests that the pressure put upon the sound kidney in these operations might be advantageously avoided by having the patient resting on a double inclined plane with the lateral pelvic surface and legs on one of the slopes, and the head and thorax on the other. In the after treatment of the above case the nitroglycerine was given with other stimulants to prevent the congestion of the remaining kidney—a congestion which always follows a nephrectomy —from being increased by the raising of arterial tension In future Brown would favour the use of strychnine with digitalis rather than with nitroglycerine. The case shows that to have a healthy kidney is not the only requisite to make nephrectomy safe. Important as it is to learn the condition of the other kidney, a sound heart may more than compensate for slight renal impairment.

[3] Med. Rec., February, 1901. [4] Monthly Homœopathic Rev., February 1901. [5] Glasgow Med. Jour., February, 1901. [6] Vide Abst. Brit. Med. Jour., February, 1901. [7] Surg. of Ann., March, 1901.

